# Plant performance of enhancing licorice with dual inoculating dark septate endophytes and *Trichoderma viride* mediated via effects on root development

**DOI:** 10.1186/s12870-020-02535-9

**Published:** 2020-07-09

**Authors:** Chao He, Wenquan Wang, Junling Hou

**Affiliations:** 1 Institute of Medicinal Plant Development, Chinese Academy of Medical Sciences & Peking Union Medical College, Beijing, 100193 China; 2 School of Chinese Pharmacy, Beijing University of Chinese Medicine, Beijing, 100029 China

**Keywords:** Licorice, Dark septate endophytes, *Trichoderma viride*, Root development, Non-host endophytes, Inoculation

## Abstract

**Background:**

This study aimed to assess whether licorice (*Glycyrrhiza uralensis*) can benefit from dual inoculation by *Trichoderma viride* and dark septate endophytes (DSE) isolated from other medicinal plants.

**Methods:**

First, we isolated and identified three DSE (*Paraboeremia putaminum*, *Scytalidium lignicola*, and *Phoma herbarum*) and *Trichoderma viride* from medicinal plants growing in farmland of China. Second, we investigated the influences of these three DSE on the performance of licorice at different *T. viride* densities (1 × 10^6^, 1 × 10^7^, and 1 × 10^8^ CFU/mL) under sterilised condition in a growth chamber.

**Results:**

Three DSE strains could colonize the roots of licorice, and they established a positive symbiosis with host plants depending on DSE species and *T. viride* densities. Inoculation of *P. putaminum* increased the root biomass, length, surface area, and root:shoot ratio. *S. lignicola* increased the root length, diameter and surface area and decreased the root:shoot ratio. *P. herbarum* increased the root biomass and surface area. *T. viride* increased the root biomass, length, and surface area. Structural equation model (SEM) analysis showed that DSE associated with *T. viride* augmented plant biomass and height, shoot branching, and root surface area. Variations in root morphology and biomass were attributed to differences in DSE species and *T. viride* density among treatments. *P. putaminum* or *P. herbarum* with low- or medium *T. viride* density and *S. lignicola* with low- or high *T. viride* density improved licorice root morphology and biomass.

**Conclusions:**

DSE isolated from other medicinal plants enhanced the root growth of licorice plants under different densities *T. viride* conditions and may also be used to promote the cultivation of medicinal plants.

## Background

Plant–microbe interactions drive plant health and the biogeochemical cycle in agricultural ecosystems [1, 2]. Plants create important habitats for the microorganisms interacting with them and deliver photosynthate to them [3]. In return, soil-borne microorganisms such as arbuscular mycorrhizal (AM) fungi, dark septate endophytes (DSE), and *Trichoderma* spp. improve plant productivity and maintain plant health. Thus, they are of particular interest in sustainable agriculture [2, 4, 5].

Dark septate endophytes (DSE) are diverse facultative biotrophic ascomycetes characterised by melanised septate hyphae and microsclerotia. They are found in the roots of > 600 different plant species [6]. The relationships between host plants and DSE range from symbiotic to parasitic depending on the particular host-symbiont combination [7]. Previous research showed that DSE inoculation stimulates the growth of medicinal plants and improves medicinal compound yield [8, 9]. Several DSE promote host plant growth by facilitating carbon, nitrogen, and phosphorus uptake [10, 11], and protecting host plants against biotic stress (pathogens) and abiotic stress (heavy metal, salt, and drought) [12–14]. *Trichoderma* spp. are common rhizosphere inhabitants that have been investigated as biological control agents, biofertilisers, and soil amendments for application in agricultural and horticultural systems [15]. *Trichoderma* spp. improve plant growth mainly by solubilising soil nutrients [16], and increasing root length and secondary root number, and upregulating phytohormones such as indoleacetic acid, cytokinin, gibberellins, and zeatin [17]. Hence, further research is required on the use of these beneficial fungi for the improvement of the growth and quality of medicinal plants.

Licorice (*Glycyrrhiza uralensis* Fisch.) is a widely distributed herbaceous perennial medicinal plant. Its roots and rhizomes are important and commonly administered medicinal materials in China and other parts of the world. It is included in the official Chinese Pharmacopoeia, a compendium of drugs compiled by the Pharmacopoeia Commission of the Ministry of Health of China. It has several pharmacological effects and biological functions because of its constituents such as glycyrrhizin and glycyrrhizic acid [8]. It also conserves water and prevents wind erosion in arid agricultural ecosystems. Moreover, its leguminous roots fix atmospheric nitrogen. Interactions between at least two different fungi and licorice have been reported [18, 19], Nevertheless, the effects of DSE combined with *Trichoderma* spp. on licorice growth have not been investigated. In earlier studies, the major DSE groups coexisted with multiple plants in various ecosystems and showed no colonisation specificity [14, 20].

In a previous study using *Trichoderma viride* for cellulose degradation, our objective was to assess the effects of DSE (*Acrocalymma vagum*, *Paraboeremia putaminum*) combined with sterilised or unsterilised organic residues containing *T. viride* on licorice growth. The combination of DSE and *T. viride* residue enhanced plant growth more effectively either agent alone [8]. Here, we used *T. viride* as a growth promoter and investigated the effects of various DSE species both alone and in combination with *T. viride* on greenhouse-raised licorice in sandy soil. We isolated DSE from the roots of *Ophiopogon japonicus* and *Lonicera japonica* on the farmlands of northern China*.* We then inoculated licorice either with the aforementioned DSE alone or in combination with various densities of *T. viride*. In this research, we endeavoured to answer the following questions: (1) What are the characteristics of DSE in the roots of *O. japonicus* and *L. japonica* from the farmlands of northern China? (2) Can these DSE colonise and influence the growth of licorice plants under artificial culture conditions? (3) Does *T. viride* density affect DSE-related symbiosis?

## Results

### Colonization characteristics and identification of DSE

Typical structure of DSE, such as dark septate hyphae and microsclerotia were observed in the roots of *O. japonicus* and *L. japonica* (Supplementary Fig. S1). Brown to yellow-brown hyphae with thick lateral walls invaded the epidermal and cortical cells (Supplementary Fig. S1A, C). Chainlike or conglomerated microsclerotia filled single or multiple cortical cells (Supplementary Fig. S1B, D).

Five and two DSE colonies isolated from *O. japonicus* and *L. japonica*, respectively, were ashen grey to dark brown (Supplementary Fig. S2). DSE1 and DSE4 produced spores but neither conidia nor reproductive structures were observed in the other isolates. A comparative analysis of the fungal sequences in the GenBank database identified *Acrocalymma vagum* (DSE1), *Paraphoma radicina* (DSE2), *Curvularia pallescens* (DSE3), *Scytalidum lignicola* (DSE4), *Paraboeremia putaminum* (DSE5), and *Phoma herbarum* (DSE6, DSE7) (Supplementary Fig. S3)*.* Based on their growth status, we selected *S. lignicola* (SL, DSE4), *P. putaminum* (PP, DSE5), and *P. herbarum* (PH, DSE6) for the pot inoculation experiments.

### Shoot morphological traits of licorice seedlings

**Fig. 1:**
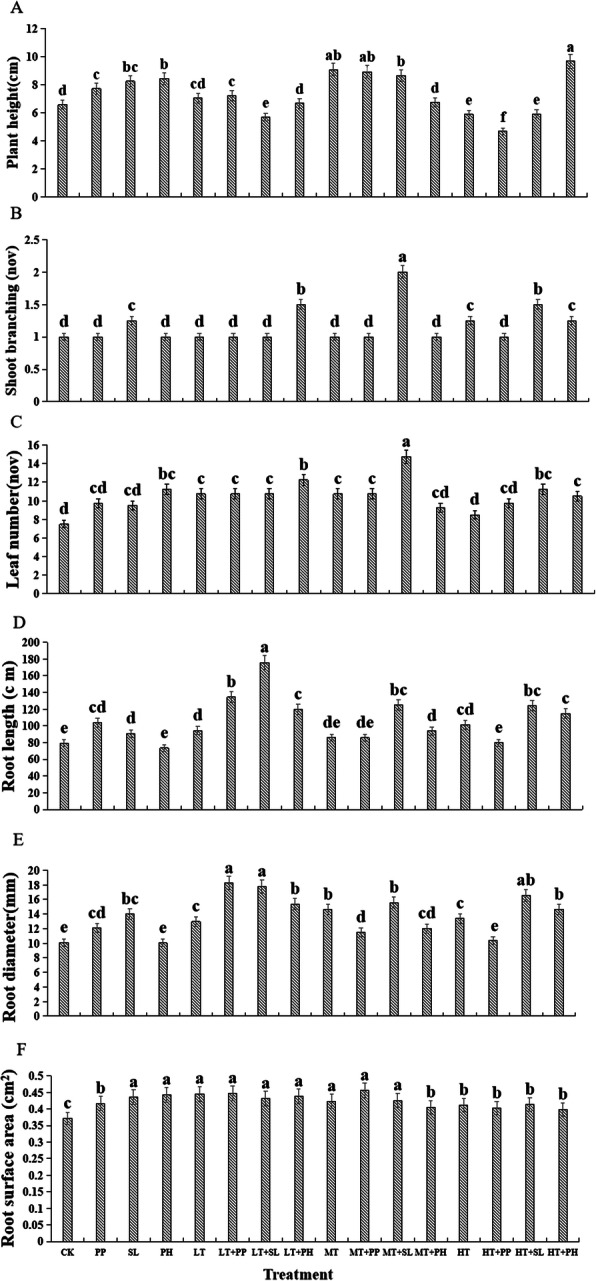
Effects of dark septate endophyte (DSE) and *Trichoderma viride* on the shoot and root morphological traits of licorice seedlings. CK indicates non-inoculated plants and no *T. viride*. PP, *P. putaminum*; SL, *S. lignicola*; PH*, P. herbarum*; LT, low *T. viride* density; MT, medium *T. viride* density; HT, high *T. viride* density; LT + PP → HT + PH, different combinations of DSE species and *T. viride* density. Different letters indicate significant differences at *P* < 0.05

After 3 months of growth, all inoculated licorice seedlings were alive, green, and healthy. The roots of all inoculated plants were colonised by DSE4, DSE5, and DSE6 (Supplementary Fig. S4). Relative to the control plants, DSE or MT inoculation significantly increased plant height whereas HT inoculation decreased it. In contrast, LT inoculation did not significantly modify plant height compared with the control plants (Fig. 1a). Only *S. lignicola* or HT inoculation significantly increased shoot branch number relative to the control plants. However, no significant differences in shoot branch number were observed between the other inoculated plants and the control plants (Fig. 1b). Compared with the control plants, *P. herbarum,* LT, and MT inoculation increased the leaf number whereas the other inoculants had no significant effect on this trait (Fig. 1c).

Interactions between DSE species and *T. viride* density were significant for plant height and shoot branch number (Table 1). Under LT conditions, *P. putaminum* inoculation increased plant height, *S. lignicola* decreased it, and *P. herbarum* had no significant effect on this characteristic compared with the control plants (Fig. 1a). Under LT conditions, only *P. herbarum* increased shoot branch and leaf numbers relative to the control plants. The other isolates had no significant impact on shoot branch or leaf number compared with the control plants (Fig. 1b, c). Under MT conditions, *P. putaminum* and *S. lignicola* increased plant height and leaf number, *S. lignicola* increased shoot branch number, and *P. herbarum* had no significant influence on plant height and shoot branch or leaf number relative to the control plants (Fig. 1a-c). Under HT conditions, compared with the control plants, *P. herbarum* increased plant height and shoot branch and leaf numbers, *S. lignicola* increased both shoot branch and leaf numbers and decreased plant height, and *P. putaminum* decreased plant height but had no effect on shoot branch or leaf numbers (Fig. 1a-c).

**Table 1:** Two-way analysis of variance for the effects of dark septate endophyte (DSE) and *Trichoderma viride* (TV) inoculation on plant biomass and morphological traits of licorice *seedlings*

	DSE		TV		DSE × TV	
	*F*	*P*	*F*	*P*	*F*	*P*
Shoot biomass (g/pot)	9.31	**< 0.001**	8.88	**< 0.001**	4.35	**< 0.001**
Root biomass (g/pot)	4.25	**0.010**	6.69	**0.001**	2.86	**0.009**
Root: shoot ratio	2.42	0.077	0.98	0.41	5.50	**< 0.001**
Plant height (cm)	0.79	0.508	4.29	**0.009**	3.04	**0.006**
Shoot branch (No.)	4.60	**0.007**	1.08	0.367	2.24	**0.034**
Leaf number (No.)	3.10	**0.035**	2.92	**0.043**	1.96	0.065
Root length (cm)	7.23	**< 0.001**	9.38	**< 0.001**	2.09	**0.048**
Root diameter (mm)	4.84	**0.006**	7.36	**< 0.001**	1.89	0.075
Root surface area (cm^2^)	1.21	0.315	4.56	**0.007**	2.39	**0.025**

Significant *P*-values are in bold

### Root morphological traits of licorice seedlings

*P. putaminum* and *S. lignicola* inoculation significantly increased licorice root length, diameter, and surface area relative to the control plants*.* However*, P. herbarum* only increased the root surface area compared with the control plants (Fig. 1d-f). Root diameter and surface area were increased at various *T. viride* densities (Fig. 1e, f). The LT and HT treatments increased the root length whereas the MT treatment had no significant impact on this trait compared with the control plants (Fig. 1d).

Under LT conditions, DSE increased the root length, diameter, and surface area relative to the control plants. Under MT conditions, DSE increased the root diameter and surface area, *S. lignicola* and *P. herbarum* increased the root length, and *P. putaminum* had no significant influence on root length compared with the control plants. Under HT conditions, DSE increased the root surface area but *S. lignicola* and *P. herbarum* only increased the root length and diameter relative to the control plants (Fig. 1d-f). The interactions between DSE species and *T. viride* density were significant only for root length and surface area (Table 1).

### Biomass production of licorice seedlings

**Fig. 2:**
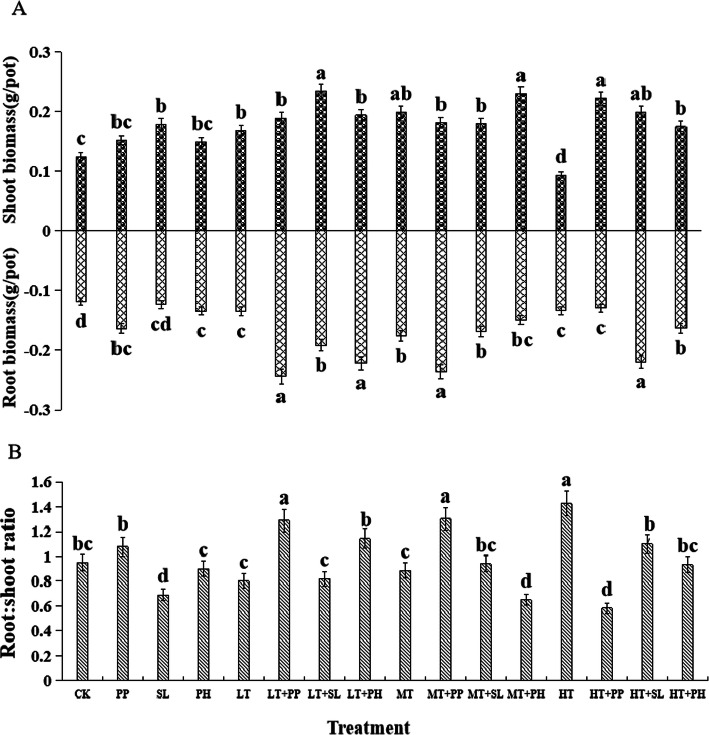
Effects of dark septate endophyte (DSE) and *Trichoderma viride* on the biomass production of licorice seedlings. CK indicates non-inoculated plants and no *T. viride*. PP, *P. putaminum*; SL, *S. lignicola*; PH*, P. herbarum*; LT, low *T. viride* density; MT, medium *T. viride* density; HT, high *T. viride* density. LT + PP → HT + PH, different combinations of DSE species and *T. viride* density. Different letters indicate significant differences at *P* < 0.05

The shoot and root biomass and the root:shoot ratio of licorice were significantly and separately influenced by DSE species and *T. viride* density (Fig. 2a). *P. putaminum* increased the root biomass and the root:shoot ratio, *S. lignicola* increased the shoot biomass and decreased the root:shoot ratio, and *P. herbarum* only increased the root biomass compared with the control plants. The LT and MT conditions increased the shoot and root biomass whereas HT increased the root biomass and the root:shoot ratio and decreased the shoot biomass relative to the control plants (Fig. 2a). There were significant positive interactions between DSE and *T. viride* with respect to shoot and root biomass. However, the interactions between DSE and *T. viride* on the root:shoot ratio varied with DSE species and *T. viride* density (Fig. 2b, Table 1).

**Fig. 3:**
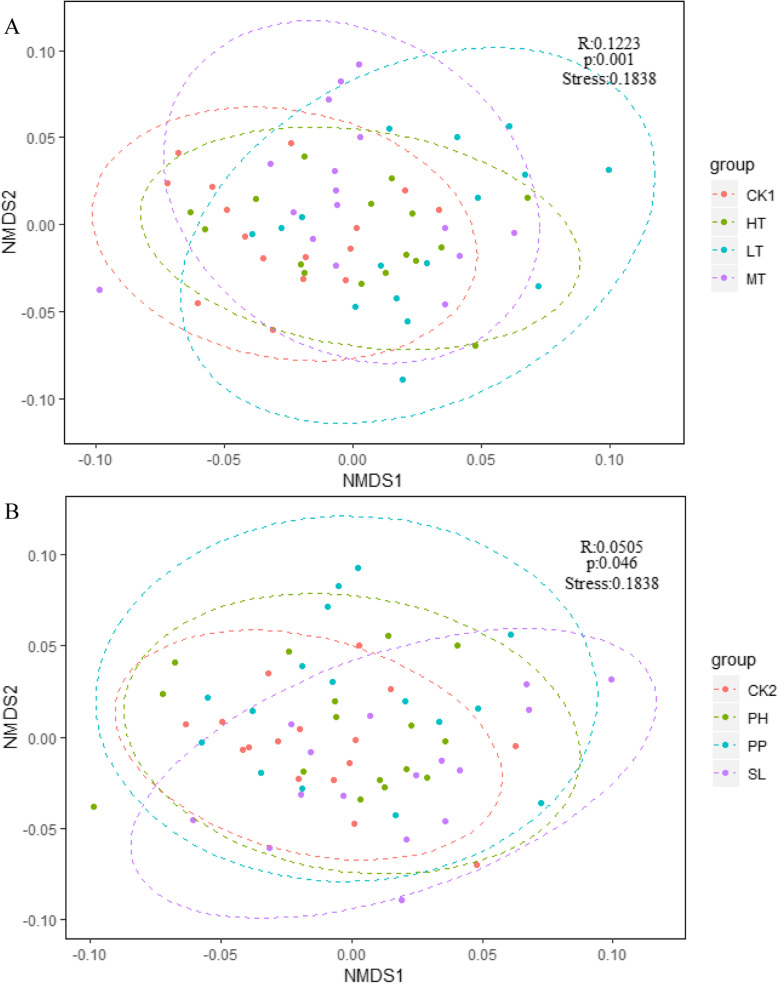
Non-metric multidimensional scaling (NMDS) ordination of the root morphological architecture of licorice seedlings under the interaction between DSE and *T. viride* density. Ellipses in the plots denote 95% confidence intervals for the centroids of root morphological architecture with *T. viride* density (**a**) and DSE species (**b**). CK1: treatments without *T. viride* with DSE; LT: low density of *T. viride* with DSE interaction; MT: moderate density of *T. viride* with DSE interaction; HT: high density of *T. viride* with DSE interaction. CK2: treatments without DSE with *T. viride*; PH: *P. herbarum* with *T. viride* interaction; PP: *P. putaminum* with *T. viride* interaction; SL: *S. lignicola* with *T. viride* interaction

Nonmetric multidimensional scaling (NMDS) and analysis of similarities (ANOSIM) indicated that licorice root morphology and biomass were significantly separated by DSE species (R = 0.0505, *P* = 0.046) and *T. viride* density (R = 0.1223, *P* = 0.001) (Fig. 3).

### Correlation analyses

**Fig. 4:**
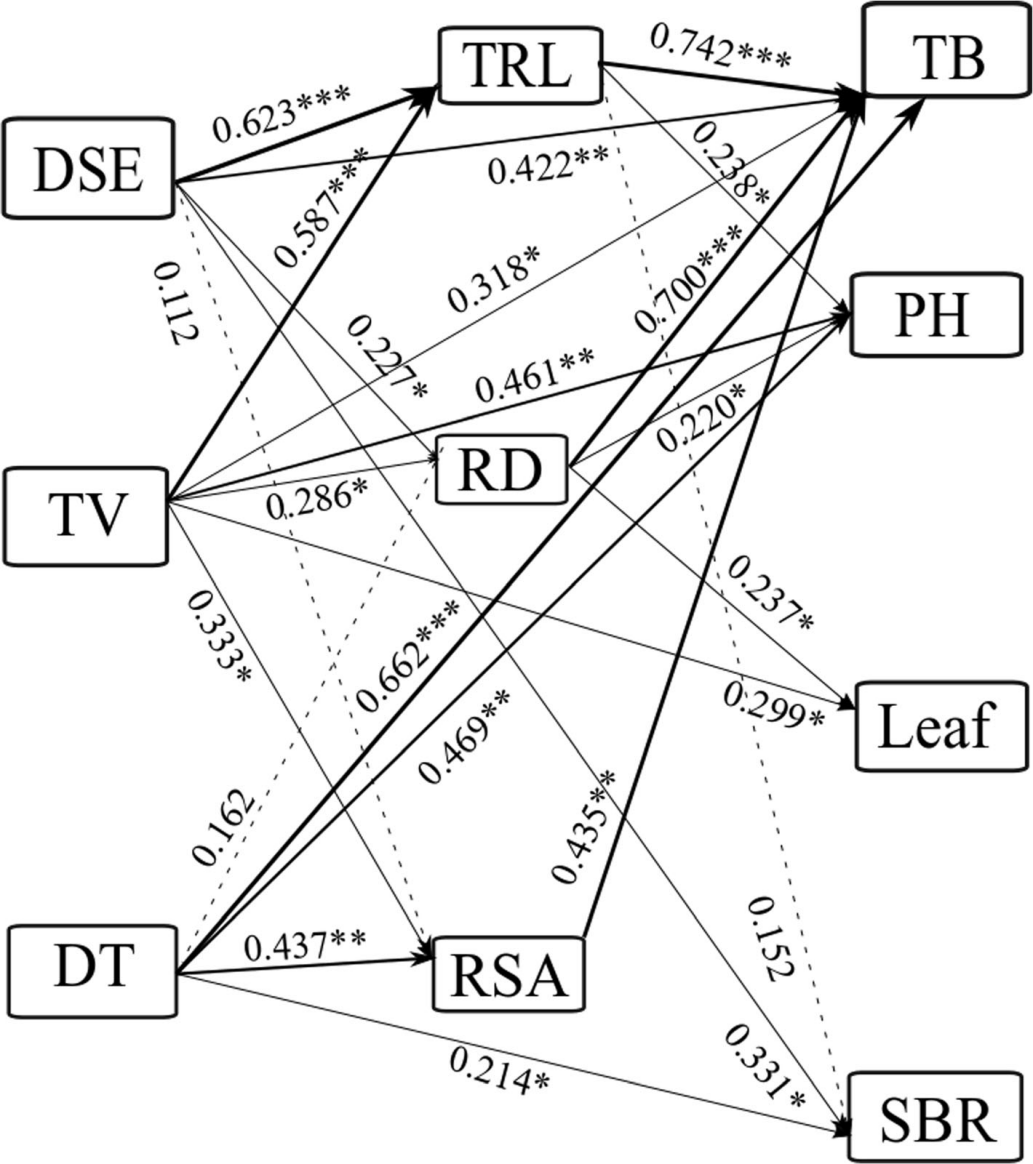
The causal relationships among DSE species, *T. viride,* root morphology and plant aboveground morphology based on structural equation model (SEM). Solid lines and dashed lines show significant and non-significant pathways, respectively. The width of the solid lines show the strength of the causal effect, and the numbers near the arrows show the standardized path coefficients (*P < 0.05, **P < 0.01, ***P < 0.001). TV = *T. viride*. DT = combination of DSE and *T. viride*. TRL = total root length. RD = root diameter. RSA = root surface area. TB = total biomass. PH = plant height. Leaf = number of leaf. SBR = branch number of shoot

A Mantel test and a structural equation model (SEM) were used to illustrate the effects of DSE, *T. viride*, and their interaction on the growth parameters of licorice. The Mantel test disclosed significant relationships among DSE, *T. viride*, plant biomass, root length, diameter, and surface area, leaf number, plant height, and shoot branching (Supplementary Table S1). We used correlation coefficients (R-values) and the SEM to quantify the relative effects of DSE, *T. viride*, the combination of DSE and *T. viride* on total root length, diameter and surface area, and plant biomass, plant height, leaf number, and shoot branching (χ^2^ = 136.933, degrees of freedom (df) = 12, *P* = 0.005, root mean square error of approximation (RMSEA) = 0.407, goodness-of fit index (GFI) = 0.750, Akaike information criterion (AIC) = 222.933). DSE had significant direct effects on root length and diameter, plant biomass, and shoot branching. *T. viride* had significant direct effects on root length, surface area, and diameter, plant biomass and height, and leaf number. The combination of DSE and *T. viride* significantly positively influenced root surface area, plant biomass and height, and shoot branching (Fig. 4).

### Variation partitioning of plant growth parameters and biomass production

**Fig. 5:**
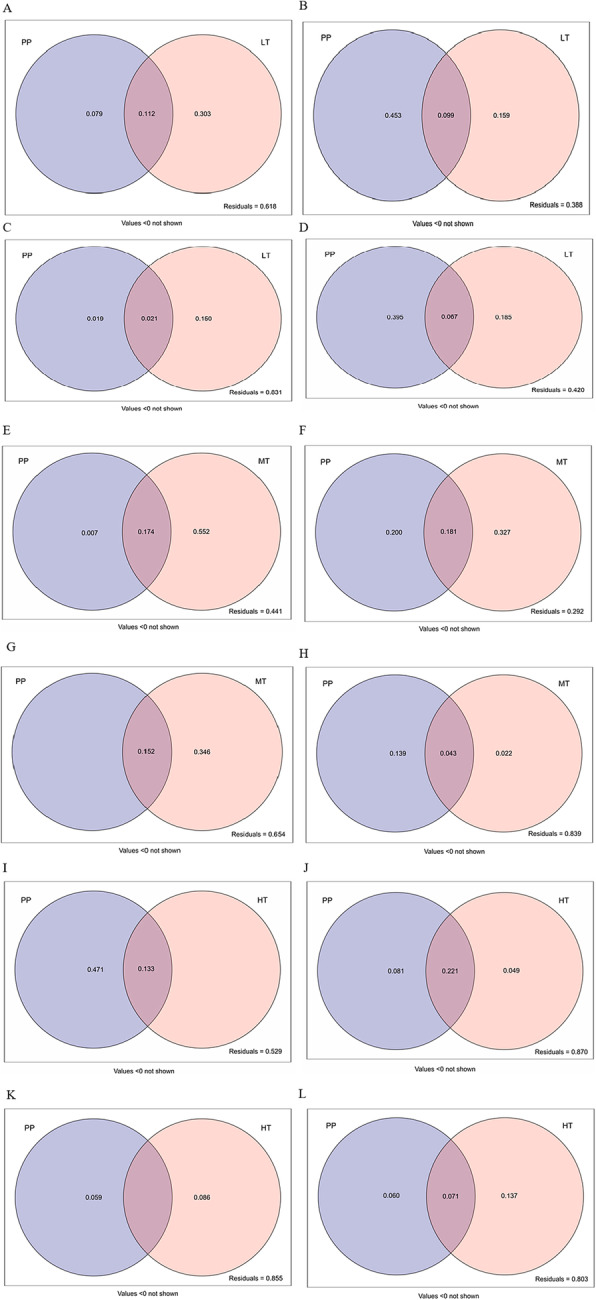
Variation partitioning of P. putaminum and T.viride of low density on plant biomass and growth traits (**a**-**d**). Variation partitioning of P. putaminum and T.viride of medium density on plant biomass and growth traits (**e**-**h**). Variation partitioning of P. putaminum and T.viride of high density on plant biomass and growth traits (**i**-**l**). PP=P. putaminum, **a**, **e**, **i**. shoot biomass; **b**, **f**, **j**. root biomass; **c**, **g**, **k**. shoot growth traits (including plant height, leaf number and branching number); **d**, **h**, **l**. root growth traits (including root length, root diameter and root surface area). Values below 0 are not shown

**Fig. 6:**
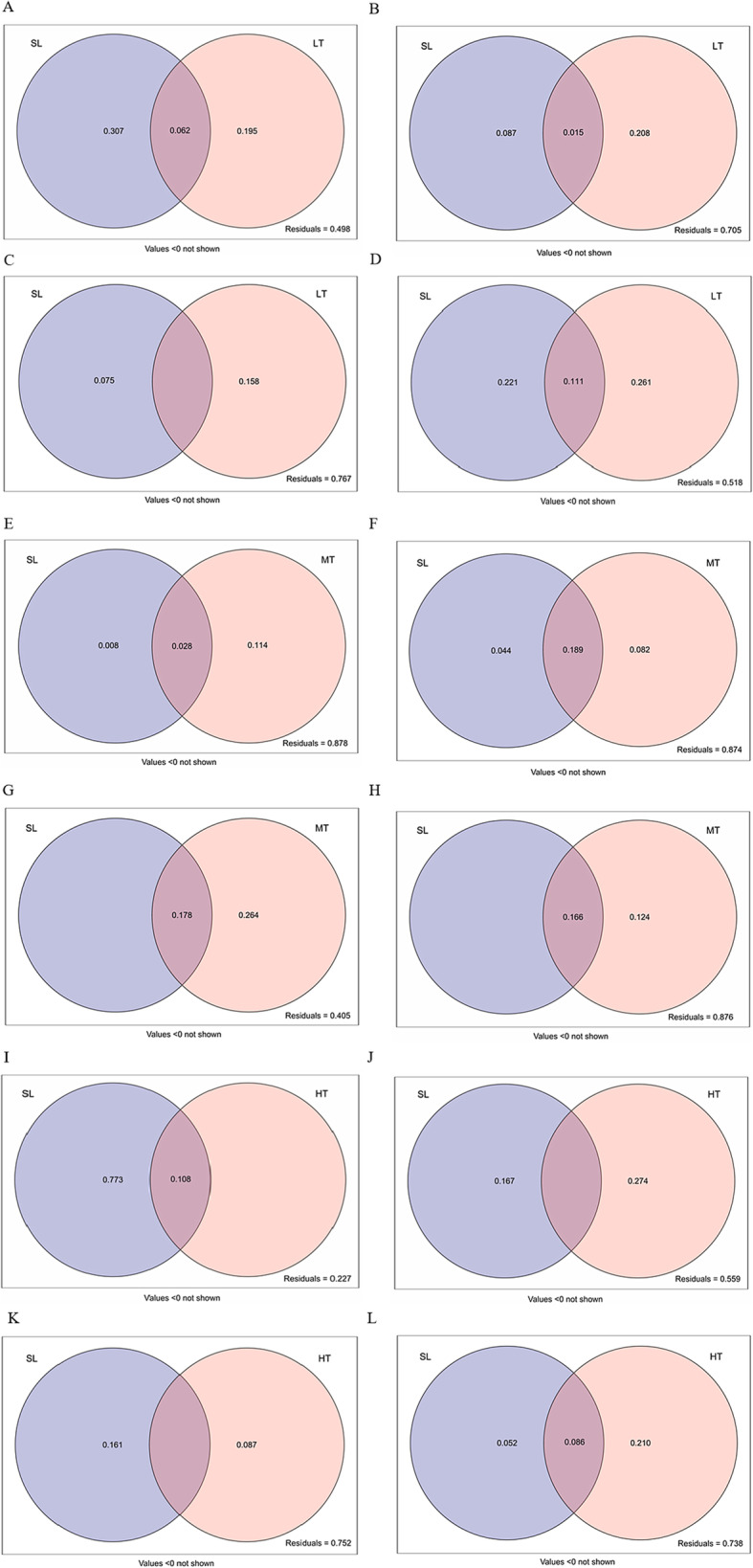
Variation partitioning of *S. lignicola* and *T.viride* of low density on plant biomass and growth traits (**a-d**). Variation partitioning of *S. lignicola* and *T.viride* of medium density on plant biomass and growth traits (**e-h**). Variation partitioning of *S. lignicola* and *T.viride* of high density on plant biomass and growth traits (**i-l**). SL = *S. lignicola*, A, E, I. shoot biomass; **b**, **f**, **j**. root biomass; **c**, **g**, **k**. shoot growth traits (including plant height, leaf number and branching number); **d**, **h**, **l**. root growth traits (including root length, root diameter and root surface area). Values below 0 are not shown

**Fig. 7:**
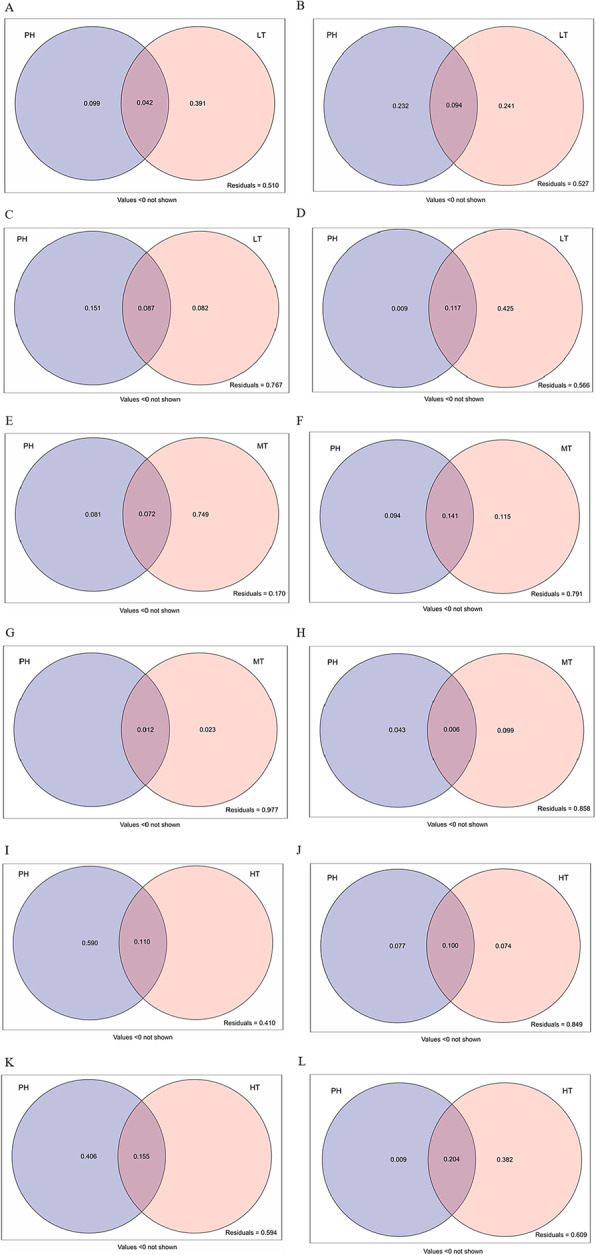
Variation partitioning of *P. herbarum* and *T.viride* of low density on plant biomass and growth traits (**a-d**). Variation partitioning of *P. herbarum* and *T.viride* of medium density on plant biomass and growth traits (**e-h**). Variation partitioning of *P. herbarum* and *T.viride* of high density on plant biomass and growth traits (**i-l**). PH=*P. herbarum*, **a**, **e**, **i**. shoot biomass; **b**, **f**, **j**. root biomass; **c**, **g**, **k**. shoot growth traits (including plant height, leaf number and branching number); **d**, **h**, **l**. root growth traits (including root length, root diameter and root surface area). Values below 0 are not shown

A variance partitioning analysis was performed to quantify the contributions of DSE species and *T. viride* density to the plant growth parameters and biomass production (Fig. 5, 6 and 7). The combination of PP and LT explained 38.2% of the variance in shoot biomass (Fig. 5a), 61.2% of the variance in root biomass (Fig. 5b), 16.9% of the variance in shoot growth traits (Fig. 5c), and 58.0% of the variance in root growth traits (Fig. 5d). The pure variances in root biomass and growth traits explained by PP were 45.3 and 39.5%, respectively, whilst LT explained 15.9 and 18.5%, respectively. The simultaneous influence of PP combined with LT on root biomass and growth traits explained 9.9 and 6.7% of the variance, respectively. The combination of PP and MT explained 55.9% of the variance in shoot biomass (Fig. 5e), 70.8% of the variance in root biomass (Fig. 5f), 34.6% of the variance in shoot growth traits (Fig. 5g), and 16.1% of the variance in root growth traits (Fig. 5h). The pure variances in root biomass and growth traits explained by PP were 20.0 and 13.9%, respectively, whilst MT explained 32.7 and 2.2%, respectively. The simultaneous influence of PP combined with MT on root biomass and growth traits explained 18.1 and 4.3% of the variance, respectively. The combination of PP and HT explained 47.1% of the variance in shoot biomass (Fig. 5i), 13.0% of the variance in root biomass (Fig. 5j), 14.5% of the variance in shoot growth traits (Fig. 5k), and 19.7% of the variance in root growth traits (Fig. 5l). The pure variances in root biomass and growth traits explained by PP were 8.1 and 6.0%, respectively, whilst HT explained 4.9 and 13.7%, respectively. The combination of PP and HT explained 22.1 and 7.1% of the variance in root biomass and growth traits, respectively.

The combination of SL and LT explained 50.2% of the variance in shoot biomass (Fig. 6a), 29.5% of the variance in root biomass (Fig. 6b), 23.3% of the variance in shoot growth traits (Fig. 6c), and 48.2% of the variance in root growth traits (Fig. 6d). The pure variances in root biomass and growth traits explained by SL were 8.7 and 22.1%, respectively, whilst LT explained 20.8 and 26.1%, respectively. The combination of SL and LT explained 5 and 11.1% of the variance in root biomass and growth traits, respectively. The combination of SL and MT explained 12.2% of the variance in shoot biomass (Fig. 6e), 12.6% of the variance in root biomass (Fig. 6f), 59.5% of the variance in shoot growth traits (Fig. 6g), and 12.4% of the variance in root growth traits (Fig. 6h). The pure variance in the root biomass explained by SL was 4.4% whilst MT explained 8.2 and 12.4% of the variance in root biomass and growth traits, respectively. The combination of SL and MT explained 18.9 and 16.6% of the variance in root biomass and growth traits, respectively. The combination of SL and HT explained 77.3% of the variance in shoot biomass (Fig. 6i), 44.1% of the variance in root biomass (Fig. 6j), 24.8% of the variance in shoot growth traits (Fig. 6k), and 26.2% of the variance in root growth traits (Fig. 6l). The pure variances in root biomass and growth traits explained by SL were 16.7 and 5.2%, respectively, whilst HT explained 27.4 and 21.0%, respectively. The combination of SL and HT explained 8.6% of the variance in shoot biomass.

The combination of PH and LT explained 49.0% of the variance in shoot biomass (Fig. 7a), 47.3% of the variance in root biomass (Fig. 7b), 23.3% of the variance in shoot growth traits (Fig. 7c), and 43.4% of the variance in root growth traits (Fig. 7d). The pure variances in root biomass and growth traits explained by PH were 23.2 and 0.9%, respectively, whilst LT explained 24.1 and 42.5%, respectively. The combination of PH and LT explained 9.4 and 11.7% of the variance in root biomass and growth traits, respectively. The combination of PH and MT explained 83.0% of the variance in shoot biomass (Fig. 7e), 20.9% of the variance in root biomass (Fig. 7f), 2.3% of the variance in shoot growth traits (Fig. 7g), and 14.2% of the variance in root growth traits (Fig. 7h). The pure variances in the root biomass and growth traits explained by PH were 9.4 and 4.3%, respectively. MT explained 11.5 and 9.9% of the variance in the root biomass and growth traits, respectively. The combination of PH and MT explained 11.4 and 0.5% of the variance in the root biomass and growth traits, respectively. The combination of PH and HT explained 59.0% of the variance in shoot biomass (Fig. 7i), 15.1% of the variance in root biomass (Fig. 7j), 40.6% of the variance in shoot growth traits (Fig. 7k), and 39.1% of the variance in root growth traits (Fig. 7l). The pure variances in the root biomass and growth traits explained by PH were 0.7 and 0.9%, respectively. HT explained 7.4 and 38.2% of the variance in root biomass and growth traits, respectively. The combination of PH and MT explained 10.0 and 20.4% of the variance in the root biomass and growth traits, respectively.

## Discussion

### Investigation and identification of DSE

DSE are common root endophytic fungi with wide host and ecogeographical ranges [13, 21]. The melanised DSE mycelia and microsclerotia characteristic in the roots of *O. japonicus* and *L. japonica* were observed in almost all root samples collected from a medicinal plant cultivation area in northern China. DSE mycelia have melanin-enriched cell walls that provide structural rigidity and protect the cells against abiotic and biotic stress [22, 23]. Microsclerotia resemble the vesicles of arbuscular mycorrhizae and are propagules or dormant structures [24]. Aaltonen [25] suggested that the melanised hyphae and microsclerotia of DSE may be essential for plant growth and survival in high-stress environments. Morphological and molecular methods identified the DSE strains isolated from *O. japonicus* and *L. japonica* roots as *Acrocalymma vagum*, *Paraphoma radicina*, *Curvularia pallescens*, *Scytalidum lignicola*, *Paraboeremia putaminum,* and *Phoma herbarum. A. vagum* and *P. putaminum* have been detected in tobacco and licorice roots [8, 9, 26]. *P. radicina* was isolated in the root surfaces of soybean [27]. *C. pallescens* was found on the surfaces of banana fruits [28], in cereals [29], and on the leaves of *Lippia sidoides* and *Humboldtia brunonis* [30, 31]. *P. herbarum* was observed in the roots of salt-stressed soybean plants [32]. These findings indicate that DSE are neither host- nor tissue-specific and may be functional in plants other than the original host [13].

### Effects of DSE or *T. viride* alone on the growth of licorice seedlings

Related studies showed that DSE inoculation had negative, neutral, or positive effects on plant growth [7, 9]. Here, all three DSE species acted as host colonisers. The DSE had significant direct effects on total root length, root diameter, plant biomass, and shoot branching in licorice*.* Relative to the control plants, *P. putaminum* increased the root biomass and root:shoot ratio, *S. lignicola* increased the shoot biomass and decreased the root:shoot ratio, and *P. herbarum* increased the root biomass. We propose that the growth-promoting efficacy of the DSE in their host plants varies with DSE species [33, 34]. Previous studies reported that *P. herbarum* promotes plant growth by producing gibberellins [32]. However, it also a causative agent of leaf spot disease in *Camellia sinensis* [35] and *Rhizoma atractylodis* [36]. *Paraboeremia* spp. (including *P. putaminum*) were recently distinguished from *Phoma* spp. *S. lignicola* was identified as a causative agent of root rot [37]. It was confirmed that the pathogenic-endophytic lifestyles of certain fungi are interchangeable [38]. There is no published information on the positive influences of *P. putaminum* and *S. lignicola* on plant growth. However, Goh et al. [39] reported that *Scytalidium parasiticum* increased plant height and leaf area in *Elaeis guineensis*. Our results corroborate the hypothesis that the pathogenic or mutualistic effects of DSE in plants may vary with DSE species and growth conditions [40, 41].

It was reported that *Trichoderma* spp*.* can be applied as a biofertiliser and plant growth promoter [41]. Here, nonmetric multidimensional scaling (NMDS) and a structural equation model (SEM) showed that *T. viride* exhibited significant direct effects on total root length, root diameter and surface area, plant biomass and height, and leaf number. Low- and medium-density *T. viride* increased shoot and root biomass. High-density *T. viride* increased root biomass and root:shoot ratio and decreased shoot biomass. Our results are consistent with those reported by Al-Hazmi and TariqJaveed [42]. They discovered that all four *Trichoderma harzianum* and *T. viride* inoculum densities increased tomato plant growth especially at the maximum density of 10^10^ spores g^− 1^ soil. Rosmana et al. [43] found that treatment with various concentrations of *Trichoderma asperellum* increased the numbers of *Theobroma cacao* buds and branches by 90.7 and 21.7%, respectively, relative to the control. Earlier research demonstrated inconsistent results for the effects of *T. harzianum* and *T. viride* on tomato plant growth [44]. Thus, the species and inoculum density of *Trichoderma* used may be key factors determining whether the relationship between this fungus and its plant host is mutualistic.

### Interactions of DSE and *T. viride* on the growth of licorice seedlings

In a previous study, we assessed the effects of combinations of DSE and organic residue on licorice*. Trichoderma viride* was only used for cellulose degradation [8]. Here, the combination of DSE and *T. viride* significantly positively influenced the root biomass and shoot biomass of licorice plants. Meanwhile, we also found that the tested DSE and *T.viride* density have different effects on the performance of different parts of licorice plants. Although fungi only colonise plant roots, certain fungal taxa interact with plant leaves and stems [45]. Two possible factors may explain the enhanced growth observed in licorice inoculated with DSE and *T. viride.* DSE and *T. viride* increase plant root growth and nutrient uptake [46, 47]. Comparatively greater root biomass is probably associated with larger numbers of lateral and secondary roots and root hairs. This adaptive root morphology augments plant nutrient uptake efficiency [48]. DSE and/or *T. viride* induced positive or neutral root system architecture responses. The production of unique bioactive compounds such as phytohormones by DSE [49, 50] and *Trichoderma* strains [51, 52], or the indirect regulation of these compounds affects growth regulator production in host plants [53–56]. Host plant phytohormone levels were not measured in the present study. However, interactions between DSE and *T. viride* significantly increased root biomass and surface area compared with the control plants. We speculate that the beneficial effects of DSE and *T. viride* on plant roots may explained by the increases in phytohormone production they promote [52, 57]. The variance partitioning analysis demonstrated persistent variance in plant biomass and growth traits. Thus, certain factors and parameters that were not measured such as DSE inoculation volume and plant culture time may have also significantly affected licorice growth.

## Conclusion

Here, we explored the associations between licorice roots and dark septate endophytes derived from the roots of *O. japonicus* and *L. japonica* grown on the farmlands of northern China. Three DSE species were effective colonizers of *O. japonicus*, *L. japonica,* and licorice roots. They had various DSE species-dependent positive effects on host plant root growth. *P. putaminum* significantly increased root biomass and root:shoot ratio and improved root morphology, *S. lignicola* improved root morphology, and *P. herbarum* increased root biomass and surface area. *T. viride* at low- and medium density increased root biomass and improved root morphology. *T. viride* at high density increased root biomass and improved root morphology. The combination of DSE and *T. viride* significantly and positively influenced root biomass, length, and surface area. Our findings substantiate the hypothesis that DSE-*T. viride* co-inoculation improves root development and nutrient absorption in host plants and could, therefore, enhance plant growth and biomass production. Licorice root is an important and valuable herbal medicine. Moreover, the whole plant reclaims drought-affected soils. Microbes living in the licorice rhizosphere form mutualistic associations with the host and help regulate its growth and stress adaptation. Future research should investigate the functions of other DSE species and evaluate the application of dual DSE-*Trichoderma* spp. inoculants in medicinal plant cultivation.

## Methods

### DSE isolation and identification

The fine roots in the rhizosphere of *O. japonicus* and *L. japonica* were collected in the Anguo Medicine Planting Site (115°20′E, 38°24′N), Hebei Province, China. Roots were surface-disinfected in 75% ethanol for 5 min and 10% NaOCl for 5 min, after which they were rinsed three times in distilled water and then dried on sterile filter paper. Finally, these segments were placed on potato dextrose agar (PDA) culture medium added with ampicillin and streptomycin sulphate. The plates were incubated at 27 °C in the dark and observed daily. To ensure that the strains were true endophytes, 200 μL of the distilled water left in the final step was coated on PDA medium and the absence of any microbial growth indicated effective surface disinfection. Colonies with dark mycelium were isolated onto PDA for observation of colony morphology and microscopic morphological characteristics [8, 9]. Totally, 26 colonies were isolated from 90 root segments. Strains with similar morphology and growth rate were grouped into the morphotypes DSE1, DSE2, DSE3, DSE4, DSE5, DSE6, and DSE7. Moreover, 3 replicates per strain were cultured at 10 °C for 2 months to induce sporulation [14, 58].

DNA was extracted from 50 mg of fresh mycelium from each colony using a genomic DNA extraction kit (Solarbio Science & Technology Co. Ltd., Beijing, China). The universal primers ITS4 (5′-TCCTCCGCTTATTGATATGC-3′) and ITS5 (5′-GGAAGTAAAAGTCGTAACAAGG-3′) were used to amplify the internal transcribed spacer (ITS) region by polymerase chain reaction (PCR) [59]. The reaction system (20 μL) included 3.5 μL DNA template, 0.5 μL of each primer (10 μM), 10 μL of 2× Es Taq Master Mix (CoWin Biosciences, Beijing, China), and 5.5 μL ddH_2_O. PCR was performed in a Life ECO™ thermocycler (Hangzhou Bioer Technology Co. Ltd., Hangzhou, China) with an initial denaturing step at 94 °C for 5 min, followed by 35 cycles of denaturation at 94 °C for 1 min, primer annealing at 55 °C for 1 min, extension at 72 °C for 1 min, and a final extension at 72 °C for 10 min [8, 60]. Finally, the PCR products were purified and sequenced, and then sequences were deposited in GenBank under accession numbers MK583545 (DSE1), MK583546 (DSE2), MK583547 (DSE3), MK583549 (DSE4), MK601233 (DSE5), MK601234 (DSE6), and MK601236 (DSE7). Clustal X (v. 1.81) was used to align the sequences. DSE phylogenetic tree were established using the maximum likelihood (ML) and Bayesian Markov chain Monte Carlo (MCMC) methods, and the ML tree was drawn with MEGA v. 6 [61] and Bayesian inference was calculated with MrBayes 3.1.2 [62]. The 4 Markov chains were run for 2 runs from random starting trees for 5 million generations, and sampling every 100 generations. The first quarter was discarded as a running in. A majority tree with the consensus rule of all remaining trees was analyzed. Branches supported by bootstrap for ML and Bayesian posterior probabilities (BPP) greater or equal than 75% (ML) and 0.95 (BPP) were confirmed as significantly supported, respectively [63]. These DSE isolates was deposited in the culture collection of the Laboratory of Endangered Species Breeding Engineering, Institute of Medicinal Plant Development, Chinese Academy of Medical Sciences and Peking Union Medical College, Beijing, China. Based on their growth status, DSE4, DSE5, and DSE6 were used for the subsequent inoculation experiments.

### Plant and *Trichoderma viride* materials

Licorice seeds were acquired from the China National Traditional Chinese Medicine Corporation of Beijing, and disinfect seeds by soaking them in 70% ethanol for 3 min, followed by 2.5% NaOCl for 10 min with agitation. Disinfected seeds were washed several times with sterile water and then aseptically planted onto water agar (10 g L^− 1^) medium in Petri dishes for germination at 27 °C [8, 14]. The *Trichoderma viride* strains were deposited in the culture collection of the Institute of Medicinal Plant Development, the Chinese Academy of Medical Sciences and Peking Union Medical College, China. The morphological characters and ITS phylogeny of *T. viride* strains were showed in Supplementary Fig. S5 and Fig. S6, and GenBank accession number was MK396066 [64, 65]. The 5 mL sterile water was placed in a Petri dish containing mature *T. viride*. The suspension was thoroughly mixed and transferred to a sterile conical flask on an ultraclean workbench [42]. The *T. viride* spore inoculum density was measured using a haemocytometer. Three *T. viride* spore concentrations were used in the subsequent inoculation experiments: low density (LT; 1 × 10^6^ CFU mL^− 1^), medium density (MT; 1 × 10^7^ CFU mL^− 1^), and high density (HT; 1 × 10 ^8^ CFU mL^− 1^).

### Inoculation assay

The inoculation experiment was performed in a growth chamber with a 14 h/10 h photoperiod, day/night temperatures of 27 °C/22 °C, and 60% mean relative humidity. The experiment lasted for 3 months and was conducted using a completely randomized factorial design (4 DSE inoculations × 3 *T. viride* densities) with 5 replicates. The 4 DSE inoculations were *Paraboeremia putaminum* (PP), *Phoma herbarum* (PH), *Scytalidium lignicola* (SL), and an uninoculated control. The 3 *T. viride* densities were low (LT), medium (MT), and high (HT). Sterilized *T. viride* spore fluid as control. Eighty experimental bottles were prepared.

About 500 g sandy soil collected from the natural habitats of licorice were placed in a glass bottle (5.5 cm in diameter at the base, 8.5 cm in diameter at the top, and 11.5 cm in height) and autoclaved at 121 °C for 2 h. Sandy soil had an organic matter content of 25.38 mg g^− 1^, available nitrogen content of 22.50 μg g^− 1^, and available phosphorus content of 1.68 μg g^− 1^. Two seedlings were planted in each sterile bottle. Two 5-mm fungal disks cut from the edge of an active DSE colony were inoculated at a 1-cm range close to the seedling roots. The uninoculated controls were treated with sterilization medium without fungi [8]. After 1 month, the seedlings were irrigated with 30 mL of various densities of *T. viride* spore fluid. The control seedlings were irrigated with 30 mL sterilized *T. viride* spore fluid. All inoculation procedures were conducted on a ultraclean workbench.

### Plant growth parameters

Before harvesting, plant height, shoot branching number, and leaf number were recorded for each plant. Shoots and roots were separately harvested from each bottle. Roots were gently washed with tap water to remove the soil. A few root samples were set aside and randomly selected to detect the DSE colonization status. Individual root sections were floated in water (nearly 1 cm deep) in a plexiglass tray and scanned using a desktop scanner (EPSON Perfection V800 Photo; Epson, Nagano, Japan). The morphological characteristics of the roots, such as root length, root diameter, and root surface area were determined using the WinRHIZO image analysis system [66]. The remaining roots and fresh shoots were dried at 70 °C for at least 48 h for calculation of the plant biomass. Total biomass production of plants was the sum of the dry weights of shoots and roots.

### DSE root colonization

Licorice roots were washed with tap water, cut into 0.5-cm-long segments, cleared in 10% KOH and then stained with 0.5% acid fuchsin. A total of 30 randomly selected 0.5-cm-long root segments in each sample were placed on slides and observed DSE colonization status under a biomicroscope [67].

### Statistical analysis

For the present experiment, two-way analysis of variance (ANOVA) was used to assess the effects of DSE, *T. viride*, and their interactions on the measured parameters. Comparisons among means were performed using Duncan’s multiple-range tests (*P* < 0.05). Nonmetric multidimensional scaling (NMDS) and an analysis of similarities (ANOSIM) test were performed using the ‘vegan’ package in R v. 3.5.3 [68] to evaluate the effects of DSE and *T. viride* on the root morphology and biomass of licorice seedlings. A Mantel test and a structural equation model (SEM) tested the effects of DSE species, *T. viride*, and their interactions on licorice morphology and biomass in the ‘ecodist’ package of R v. 3.2.2 [69]. Variance partitioning was used to assess the magnitude of influence of each factor on the plant growth parameters and biomass production. SPSS 21.0, AMOS v. 21.0 (Maximum likelihood), Canoco v. 4.5, RStudio ‘vegan’ package [70], and KaleidaGraph v. 4.5 were used for analyzing and graph drawing.

## Supplementary information

**Additional file 1: Figure S1.** Dark septate endophytes (DSE) associated with the roots of O. japonicus (A, B) and L. japonica (C, D). (A, C) DSE hyphae; (B, D) DSE microsclerotia. Arrows indicate the following: Hy, DSE hyphae; M, DSE microsclerotia. Bars = 50 μm. **Figure S2.** (A–G) Colonies of dark septate endophyte (DSE) isolated from the roots of O. japonicus (A–C, E, F) and L. japonica (D, G) on PDA medium. (a–g) Microscopic morphology of DSE fungi. Bars = 50 μm. (A, a) DSE 1; (B, b) DSE 2; (C, c) DSE 3; (D, d) DSE 4; (E, e) DSE 5; (F, f) DSE 6; (G, g) DSE 7. Arrows indicate the following: Hy, DSE hyphae; S, DSE spores. **Figure S3.** Maximum parsimony tree generated from ITS region sequences of the DSE strains and their closest matches, followed by GenBank accession number. Parsimony bootstrap proportions (before the/) higher than 50 % and Bayesian posterior probabilities (after the/) more than 0.95 were indicated along branches. **Figure S4.** Colonization by three dark septate endophyte (DSE) strains in the roots of inoculated licorice seedlings after 3 months. Hy, DSE hyphae; M, DSE microsclerotia. Bars = 50 μm. (A, B) P. putaminum; (C, D) S. lignicola; (E, F) P. herbarum. **Figure S5.** Colony morphology (A, B) and conidiophore (C) and conidium (D) of Trichoderma viride strain on potato dextrose agar. Arrows indicate the following: S, spore of T. viride. Bar = 20 μm. **Figure S6.** Maximum parsimony tree generated from ITS region sequences of Trichoderma strains and their closest matches, followed by GenBank accession number. **Table S1.** Mantel tests showing correlationship (R values) between DSE, T.viride, total root length, root diameter, root surface area, total biomass, plant height, number of leaf and shoot branching.

## Data Availability

All data generated or analyzed during this study are included in this manuscript and its supplementary information files, and the datasets used and/or analysed during the current study are available from the corresponding author on reasonable request.

## Abbreviations

DSE: Dark septate endophytes
*T. viride* or TV: *Trichoderma viride*
*S. lignicola* or SL: *Scytalidium lignicola*
*P.putaminum* or PP: *Paraboeremia putaminum*
*P.herbarum* or PH: *Phoma herbarum*
AM: Arbuscular mycorrhiza
*G. uralensis*: *Glycyrrhiza uralensis*
*O. japonicus*: *Ophiopogon japonicus*
*L. japonica*: *Lonicera japonica*
PDA: Potato dextrose agar
ITS: Internal transcribed spacer
PCR: Polymerase chain reaction
MCMC: Markov chain Monte Carlo
ML: Maximum likelihood
BPP: Bayesian posterior probabilities
LT: Low density of *Trichoderma viride*
MT: Medium density of *Trichoderma viride*
HT: High density of *Trichoderma viride*
KOH: Potassium hydroxide
NMDS: Nonmetric multidimensional scaling
SEM: Structural equation model
RMSEA: Root mean square error of approximation
GFI: Goodness-of fit index
AIC: Akaike information criterion
