# Supplementary material for: Plant performance of enhancing licorice with dual inoculating dark septate endophytes and Trichoderma viride mediated via effects on root development
Source: BMC Plant Biol. 2020 Jul 9;20:325. doi: 10.1186/s12870-020-02535-9 (PMC7346674; doi:10.1186/s12870-020-02535-9)
Supplement: Supplementary file 1 — Additional file 1: Figure S1. Dark septate endophytes (DSE) associated with the roots of O. japonicus (A, B) and L. japonica (C, D). (A, C) DSE hyphae; (B, D) DSE microsclerotia. Arrows indicate the following: Hy, DSE hyphae; M, DSE microsclerotia. Bars = 50 μm. Figure S2. (A–G) Colonies of dark septate endophyte (DSE) isolated from the roots of O. japonicus (A–C, E, F) and L. japonica (D, G) on PDA medium. (a–g) Microscopic morphology of DSE fungi. Bars = 50 μm. (A, a) DSE 1; (B, b) DSE 2; (C, c) DSE 3; (D, d) DSE 4; (E, e) DSE 5; (F, f) DSE 6; (G, g) DSE 7. Arrows indicate the following: Hy, DSE hyphae; S, DSE spores. Figure S3. Maximum parsimony tree generated from ITS region sequences of the DSE strains and their closest matches, followed by GenBank accession number. Parsimony bootstrap proportions (before the/) higher than 50 % and Bayesian posterior probabilities (after the/) more than 0.95 were indicated along branches. Figure S4. Colonization by three dark septate endophyte (DSE) strains in the roots of inoculated licorice seedlings after 3 months. Hy, DSE hyphae; M, DSE microsclerotia. Bars = 50 μm. (A, B) P. putaminum; (C, D) S. lignicola; (E, F) P. herbarum. Figure S5. Colony morphology (A, B) and conidiophore (C) and conidium (D) of Trichoderma viride strain on potato dextrose agar. Arrows indicate the following: S, spore of T. viride. Bar = 20 μm. Figure S6. Maximum parsimony tree generated from ITS region sequences of Trichoderma strains and their closest matches, followed by GenBank accession number. Table S1. Mantel tests showing correlationship (R values) between DSE, T.viride, total root length, root diameter, root surface area, total biomass, plant height, number of leaf and shoot branching. [file 12870_2020_2535_MOESM1_ESM.doc]

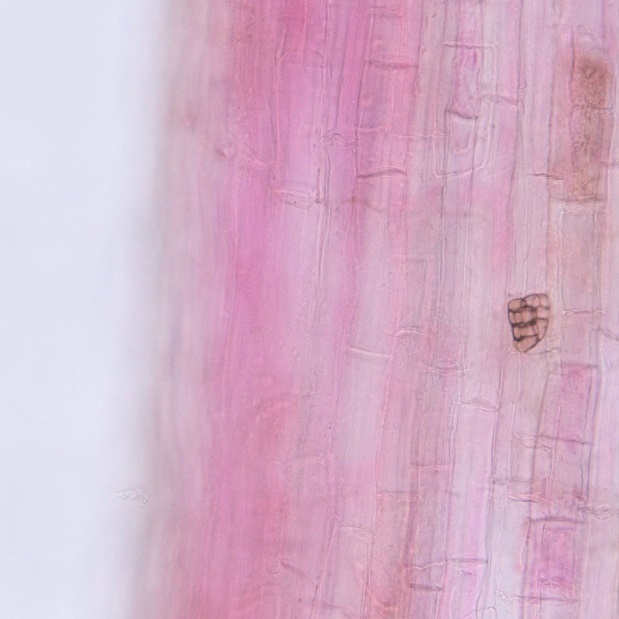


**50μm**

**B**

**M**


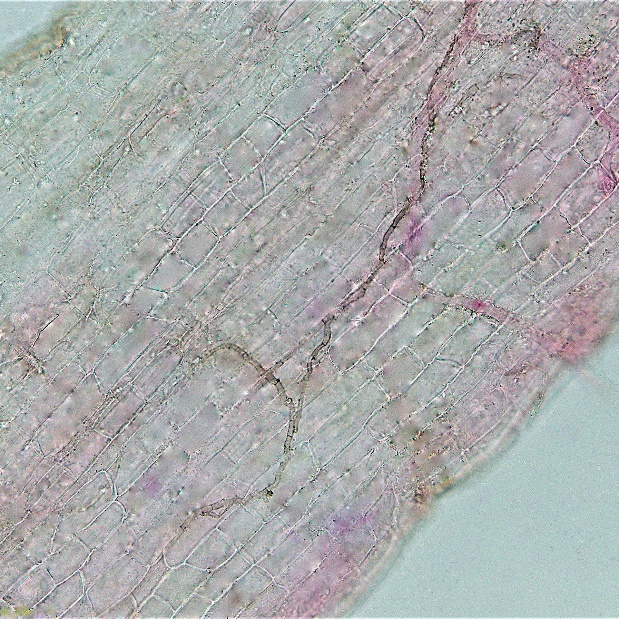


**50μm**

**Hy**

**A**


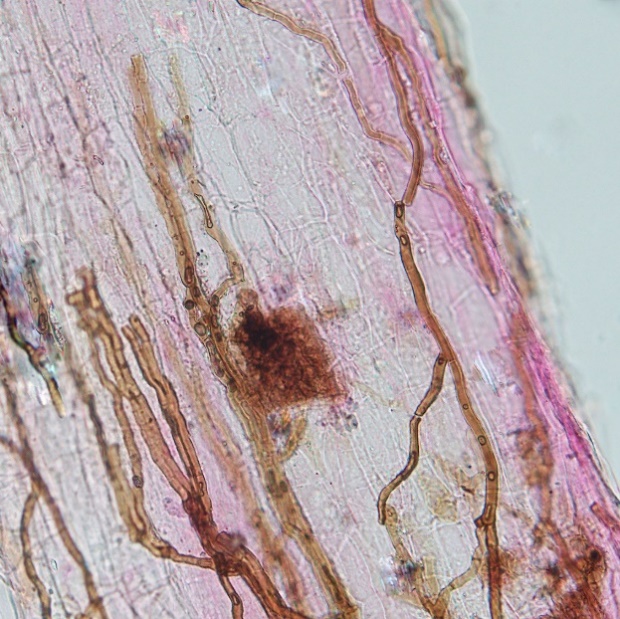


**Hy**

**C**

**50μm**


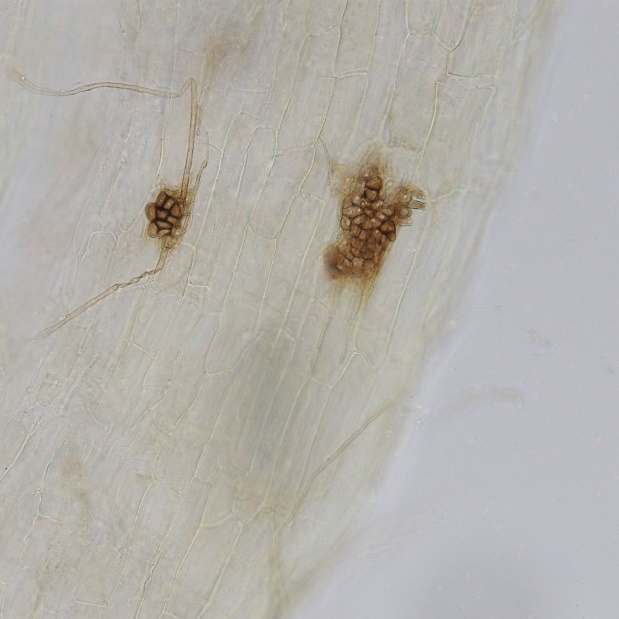


**M**

**D**

**50μm**

Fig. S1 Dark septate endophytes (DSE) associated with the roots of *O. japonicus* (A, B) and *L. japonica* (C, D). (A, C) DSE hyphae; (B, D) DSE microsclerotia. Arrows indicate the following: Hy, DSE hyphae; M, DSE microsclerotia. Bars = 50 μm.


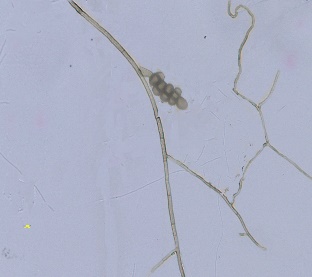

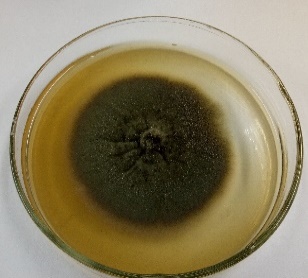


**E**


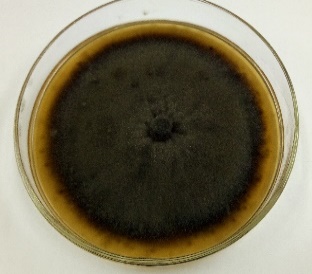


**F**


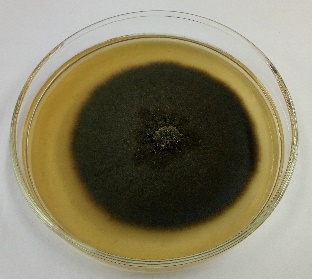


**G**


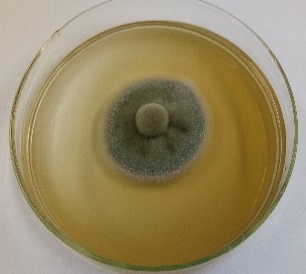


**A**


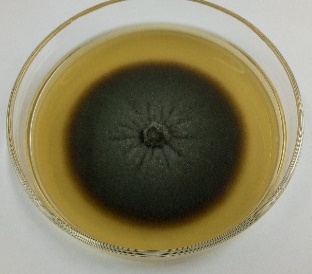


**B**


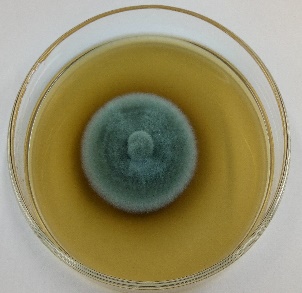


**C**


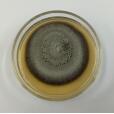


**D**

**a**


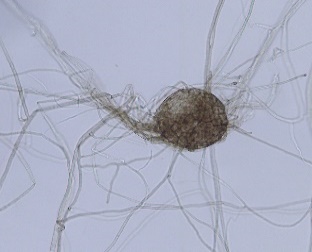


**b**


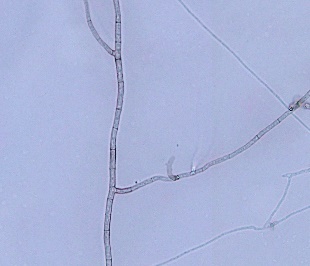


**c**


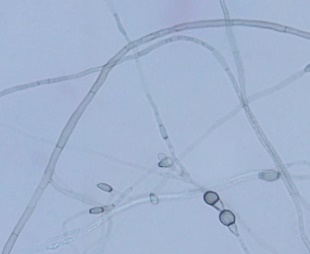


**d**


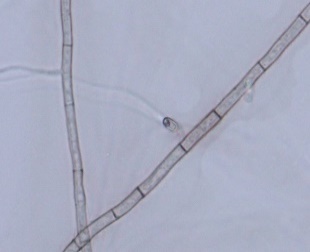


**e**


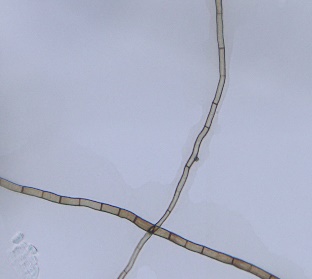


**f**


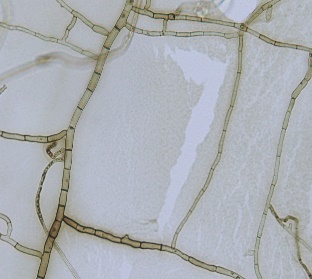


**g**

**S**

**Hy**

**Hy**

**Hy**

**Hy**

**S**

**Hy**

**Hy**

**Hy**

**50μm**

**50μm**

**50μm**

**50μm**

**50μm**

**50μm**

**50μm**

Fig. S2 (A–G) Colonies of dark septate endophyte (DSE) isolated from the roots of *O. japonicus* (A–C, E, F) and *L. japonica* (D, G) on PDA medium. (a–g) Microscopic morphology of DSE fungi. Bars = 50 μm. (A, a) DSE 1; (B, b) DSE 2; (C, c) DSE 3; (D, d) DSE 4; (E, e) DSE 5; (F, f) DSE 6; (G, g) DSE 7. Arrows indicate the following: Hy, DSE hyphae; S, DSE spores.


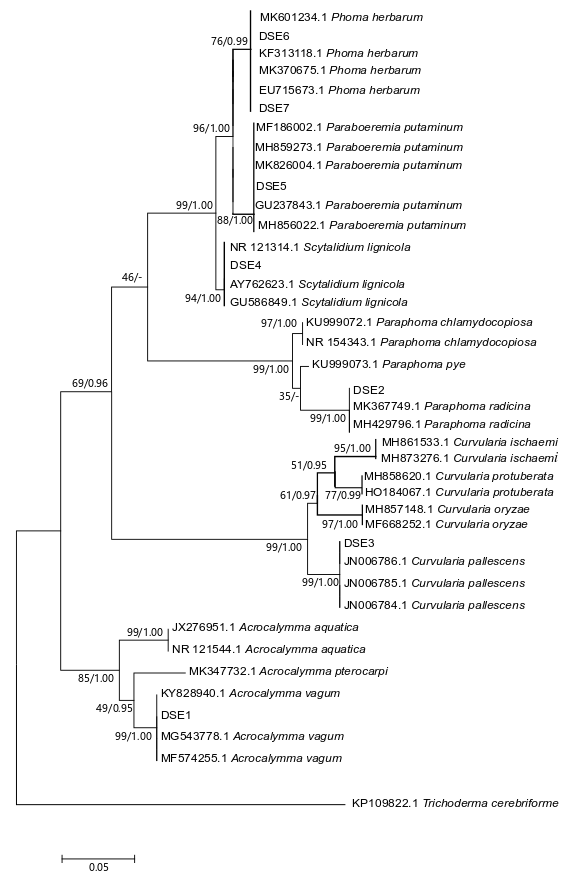
Fig. S3 Maximum parsimony tree generated from ITS region sequences of the DSE strains and their closest matches, followed by GenBank accession number. Parsimony bootstrap proportions (before the/) higher than 50 % and Bayesian posterior probabilities (after the/) more than 0.95 were indicated along branches


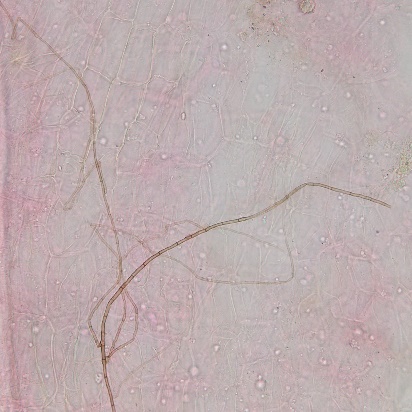


**Hy**

**A**


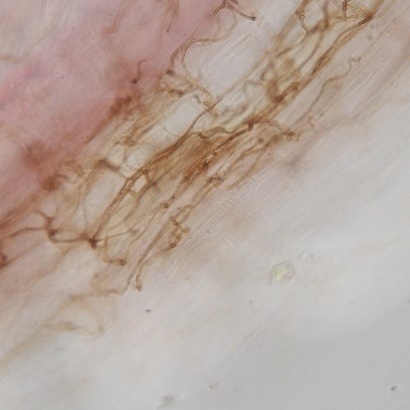


**Hy**

**C**


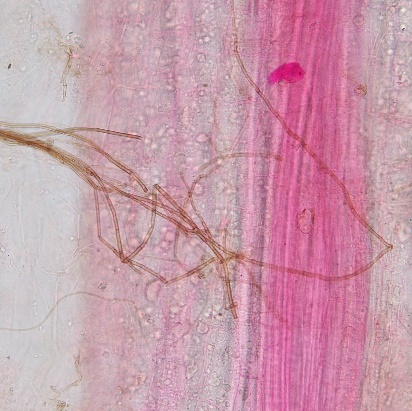


**Hy**

**E**


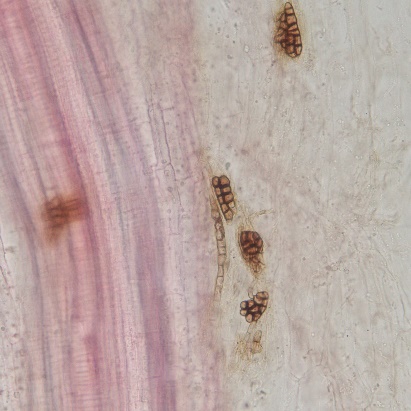


**M**

**B**


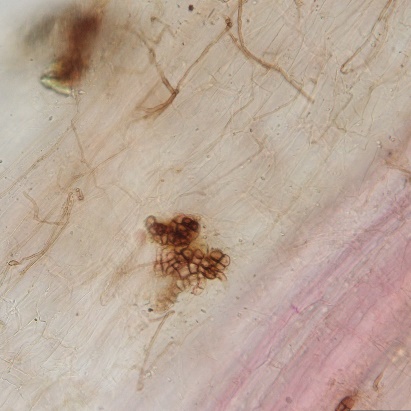


**M**

**Hy**

**D**


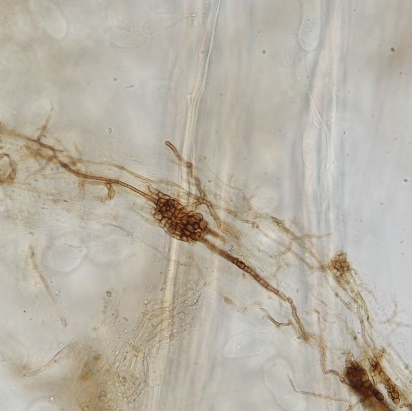


**M**

**Hy**

**F**

**50μm**

**50μm**

**50μm**

**50μm**

**50μm**

**50μm**

Fig. S4 Colonization by three dark septate endophyte (DSE) strains in the roots of inoculated licorice seedlings after 3 months. Hy, DSE hyphae; M, DSE microsclerotia. Bars = 50 μm. (A, B) *P. putaminum*; (C, D) *S. lignicola*; (E, F) *P. herbarum.*


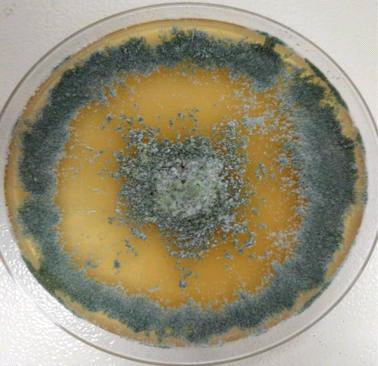

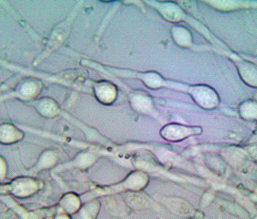

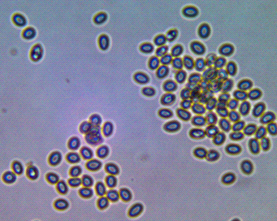

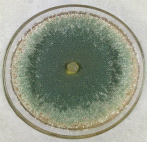


**A**

**B**

**C**

**D**

**S**

**S**

**20µm**

**20µm**

Fig. S5 Colony morphology (A, B) and conidiophore (C) and conidium (D) of *Trichoderma viride* strain on potato dextrose agar. Arrows indicate the following: S, spore of *T. viride*. Bar = 20 µm.


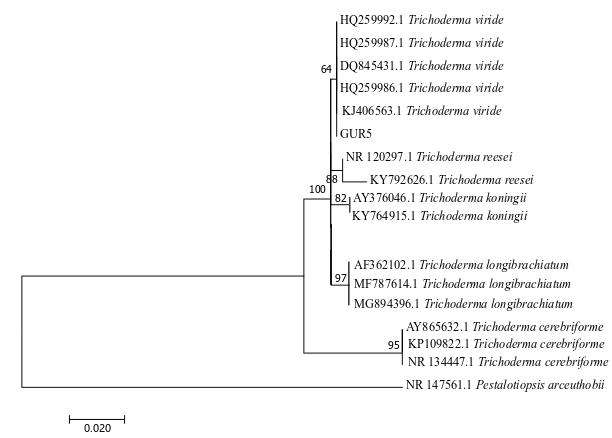


Fig. S6 Maximum parsimony tree generated from ITS region sequences of *Trichoderma* strains and their closest matches, followed by GenBank accession number.

**Table S1** Mantel tests showing correlationship (R values) between DSE, *T.viride*, total root length, root diameter, root surface area, total biomass, plant height, number of leaf and shoot branching

| Variable | DSE | TV | DT | TRL | RD | RSA | TB | PH | Leaf | SBR |
| --- | --- | --- | --- | --- | --- | --- | --- | --- | --- | --- |
| DSE | 1 |  |  |  |  |  |  |  |  |  |
| TV | -0.027 | 1 |  |  |  |  |  |  |  |  |
| DT | 0.381*** | 0.284*** | 1 |  |  |  |  |  |  |  |
| TRL | 0.299*** | 0.333*** | 0.120 | 1 |  |  |  |  |  |  |
| RD | 0.158* | 0.166* | 0.092 | 0.388*** | 1 |  |  |  |  |  |
| RSA | 0.085 | 0.159* | 0.248** | 0.265*** | 0.111 | 1 |  |  |  |  |
| TB | 0.215** | 0.178* | 0.302*** | 0.271*** | 0.192** | 0.227** | 1 |  |  |  |
| PH | 0.118 | 0.195* | 0.222** | 0.215* | 0.157* | -0.037 | 0.089 | 1 |  |  |
| Leaf | 0.105 | 0.143* | 0.064 | 0.016 | 0.185* | 0.005 | 0.187* | 0.061 | 1 |  |
| SBR | 0.188* | 0.072 | 0.153* | 0.055 | -0.006 | -0.078 | 0.029 | 0.164* | 0.228** | 1 |
